# Structured self monitoring of blood glucose in Iranian people with type 2 diabetes; A cost consequence analysis

**DOI:** 10.1186/2008-2231-20-32

**Published:** 2012-09-10

**Authors:** Rokhsareh Aghili, Mohammad E Khamseh, Mojtaba Malek, Shahin Yarahmadi, Amir Farshchi

**Affiliations:** 1 Endocrine Research Center (Firouzgar), Institute of Endocrinology and Metabolism (Hemmat Campus), Tehran University of Medical Sciences, Tehran, Iran; 2 Endocrinology and Metabolic Office, Center for Disease Control, Ministry of Health and Medical Education, Tehran, Iran; 3 Department of Pharmacoeconomics and Pharmaceutical Administration, School of Pharmacy, Tehran University of Medical Sciences, Tehran, Iran

**Keywords:** Structured self-monitoring of blood glucose, Diabetes, Cost analysis

## Abstract

**Background:**

Self-Monitoring of Blood Glucose (SMBG) is considered as a key factor in management of people with diabetes which is a growing and cost demanding health problem. The purpose of this study was to investigate the effect of comprehensive patient management using structured SMBG on metabolic control as well as its cost consequence analysis.

**Methods:**

Sixty subjects were recruited in an observational study for a period of 6 months. They were provided with the ACCU-CHEK 360° View tool to fill in the values of the 7-point blood glucose profiles in three consecutive days during the study on a monthly basis. Changes in metabolic control were assessed by HbA1c and lipid profile measurement at the beginning and at the end of the study. In addition, cost consequence analysis was done considering different level of health care professionals with or without insurance coverage. The Average Cost Effectiveness Ratio (ACER) as well as Cost saving analysis were calculated and compared.

**Results:**

The analysis showed significant reduction in HbA1c during the 6-month period in all subjects (P = 0.000). Furthermore, a positive effect was observed on lipid profile. The cost of endocrinologist’s visit in private sector was estimated to be 265.76 USD while this figure was149.15 USD for general practitioner in public sector with insurance coverage. Total complications and mortality cost saving was 154.8 USD. The lowest ACER was calculated for intervention with general practitioner in public sector with insurance coverage.

**Conclusion:**

Structured SMBG results in significant improvement of glycemic status. Moreover, it is more cost saving in public sector with insurance coverage. It seems that general practitioner visits with insurance coverage is the most affordable option for people with type 2 diabetes.

## Introduction

Diabetes is a complex and growing health problem with significant social and economic burden [1]. In 2011, it was estimated that there were 366 million adults with diabetes throughout the world an increase of more than 120 million since 2007 [2]. By 2030, the number of adults with diabetes is expected to rise to 552 million [2]. The prevalence of type 2 diabetes mellitus (T2DM) in Iran was reported to be 7.7% in people younger than 65 years [3].

There are many risk factors for development of diabetes [4]. Diabetes is associated with a decrease in life expectancy [2] and people with diabetes are at increased risk for developing various cost-demanding complications [5]. Therefore, diabetes has a profound impact on the physical, psychological, and financial well-being of individuals, their families, and the society [5].

The benefits of strict glycemic control on reducing the risk of micro and macrovascular complications are well documented [6-8]. For example, the United Kingdom Prospective Diabetes Study (UKPDS) demonstrated that every percentage point decrease in HbA_1c_ is associated with significant reduction in diabetes related deaths (21%), myocardial infarction (14%) and microvascular disease (37%) [7]. Self-monitoring of blood glucose (SMBG) enables people with diabetes to modify their behavior and adjust their treatment according to the results obtained by blood glucose monitoring [9]. It also helps them to be active in diabetes educational programs through a deep understanding of the patterns provided by SMBG [9,10]. However, the cost of SMBG is a major problem. It was reported to be around £90 million in the UK in 2001 [8].

Since the first description of structured SMBG [11] there has been controversial issues regarding its indications, frequencies and cost effectiveness in people with type 2 diabetes [12]. The results of several studies supported the cost effectiveness of structured SMBG for glycemic control in diabetic people [13,14]; however, other studies did not support the issue [15,16]. The role of the general practitioner as gatekeepers between primary and secondary care has great importance in cost-effectiveness of the intervention [17,18]. In addition, health insurance coverage is the other noteworthy factor to cost effective healthcare [19].

The aim of this observational study was to investigate the effect of comprehensive patient management using structured SMBG on metabolic control as well as its cost consequence analysis considering different level of health care professionals with or without insurance coverage in people with type 2 diabetes.

## Methods

We performed a 6-month observational study, exploring the effect of a comprehensive approach in diabetes management. People with uncontrolled type 2 diabetes (HbA1c ≥ 8) were recruited from the outpatient diabetes services at Institute of Endocrinology and Metabolism (IEM).

Exclusion criteria included pregnancy, previous use of self-monitoring of blood glucose, and far advanced complications.

At enrollment, basic education was provided to the eligible subjects. People participated in two face-to-face educational sessions; ACCU-CHEK® Assist was used for this purpose. All participants received education about the meter device as well as essential instructions in order to record the results. Instructions were provided how to use the ACCU-CHEK® 360° View paper tool, and to fill in the values of the 7-point blood glucose profiles in three consecutive days, for 6 months during the study on a monthly basis. Seven-point SMBG includes three pre-meals, three post-meals, and bedtime blood glucose values during each day.

All people were supplied with ACCU-CHEK® Performa glucometers, strips, lancets, and lancing devices, plus the 360° View forms and were asked to record their blood glucose values at the end of each month on 3 consecutive days. During the study, physicians were free to adjust the medical treatment of all diabetic people when needed.

Changes in metabolic control were assessed by HbA1c and lipid profile measurement at enrollment and at the end of the study. HbA1c was measured using ion exchange chromatography (DS5 Analyser, Drew Scientific limited, Cumbria, United Kingdom).

Ethical approval was granted from the ethics board at IEM.

All data are presented as mean ± standard deviation (SD). Significant differences in general characteristics were determined by Chi-square and Student’s t-test. SPSS for Windows (Version 19; SPSS Inc., Chicago, IL) was used for data analyses and p values < 0.05 were considered statistically significant.

### Cost consequence analysis

#### Costs

We calculated direct costs of intervention. Resources used as costs of the intervention were: patient education, laboratory tests, glucometer price, test strips and physician visits during the study. We considered three main levels of healthcare professionals to calculate and compare the direct costs of the intervention in public and private sectors. All tariffs data were gathered from the official website of Ministry of Health (MoH) of Iran [20]. Costs from health provider perspective, were converted from Iranian Rials (IRR) into USA dollar (USD) at an official exchange rate of 9,920 IRR/1USD 2010 [21] to have an international comparison.

#### Average cost effectiveness analysis

Average changes in HbA1c from beginning to the end of the study were considered as clinical outcome (effect) and the Average Cost Effectiveness Ratio (ACER) was calculated according to the below formula for each patient: ACER = Cost/ Outcome [22].

The comparison of ACERs was held afterwards. We used sensitivity analyses to examine the effects of costs of these types of visits on the main results.

#### Cost saving

With this technique, we evaluated cost saving related outcome improvement in SMBG intervention. Changing in HbA1c mentioned as standard indicator of intervention efficacy and linked to money saved according to two previously published evidences. Health care expenditures attributed to T2DM complications were extracted from the study conducted by Javanbakht et al. [23] and were linked to Stratton’s study [7] to estimate the monetary benefits of changes in HbA1c. Finally, Cost saving for each alternative approaches were reported.

## Results

Sixty subjects were included in this study. Thirty three (55%) were female and 27 (45%) were male. The mean age was 52.7 [±7.9 SD], and the mean duration of diabetes was 9.0 [±7.1 SD] years. Table 1 illustrates baseline characteristics of the participants.

**Table 1:** Baseline characteristics of the participants

**Variables**	
Age	52.7 (± 7.9)
Duration of DM	9.0 (± 7.1)
Gender	
Female	33 (55%)
Male	27 (45%)
Diabetes treatment	
Diet ± Oral agent	45 (75%)
Diet ± Insulin	10 (16.70%)
Insulin ± Oral agent ± Diet	5 (8.30%)

Mean ± SD are shown for continuous variables and % is shown for categorical variables.

DM; Diabetes Mellitus.

HbA1c as the primary end point was improved significantly from 10.2% to 8.5% (P = 0.000). In addition, we observed a positive effect of structured SMBG on BMI, waist circumference, blood pressure as well as metabolic outcomes (Tables 2 and 3).

**Table 2:** Effect of Structured SMBG on BMI, waist circumference and blood pressure

	**Baseline (Mean ± SD)**	**End of study (Mean ± SD)**	**Mean Difference**	**CI**	**p-value**
BMI (kg/m^2^)	28.9 (± 5.0)	28.6 (±1.1)	-0.3	-0.07, 0.72	NS
Waist Circumference (cm)	98.9 (± 11.2)	97.2 (± 5.2)	-1.7	-2.71, 2.32	NS
Systolic Blood Pressure (mmHg)	124.6 (± 15.5)	122.7 (± 15.2)	-1.9	0.21, 10.87	0.042
Diastolic Blood Pressure (mmHg)	79.0 (± 5.4)	78.3 (± 8.2)	-0.7	0.16, 4.39	0.035

Data are shown as Mean ± SD.

P < 0.05 is considered as statistically significant.

NS; Not Significant.

BMI; Body Mass Index.

**Table 3:** Effect of structured SMBG on metabolic variables

	**Baseline (Mean ± SD)**	**End of study (Mean ± SD)**	**Mean Difference**	**CI**	**p-value**
HbA1c	10.2 (± 1.6)	8.5 (± 2.0)	-1.7	1.10, 2.30	0.000
HDL	39.9 (± 8.9)	43.0 (± 8.6)	3.1	-5.69, -0.52	0.019
LDL	86.7 (± 28.9)	83.9 (± 27.2)	-2.8	-5.35, 10.95	NS
TG	129.6 (± 62.6)	122.4 (± 43.6)	-7.2	-5.91, 20.26	NS
HDL/LDL ratio	0.5 (± 0.2)	0.6 (± 0.1)	0.1	-0.09, -0.014	0.009

Data are shown as Mean ± SD.

P < 0.05 is considered as statistically significant.

NS; Not Significant.

### Consequences cost analysis

#### Costs

According to our data from cost resources and also MoH defined tariffs, different hypothesis of costs were compared. Table 4 shows the direct cost of intervention in public and private sectors. Total costs per patient with different type of visit and lab test tariffs are presented in Table 5. The highest and the lowest cost were related to endocrinologist in private sector (265.76 USD) and general practitioner in public sector with insurance (149.15) respectively.

**Table 4:** Direct costs of the intervention in public and private sectors

**Items**	**Count**	**Unit cost (USD)**	**Total cost (USD)**	**Cost per patient (USD)**
Patient education	4	221.77	886.72	14.78
Lab tests				
Private	120	15.12	1814.4	30.24
Public				
- Insured	120	1.71	205.2	3.42
- Uninsured	120	6.86	823.2	13.72
Glucometer device	60	58.47	3508.2	58.47
Test Strips and lancets	126	29.03	3657.78	60.96
Educational tool	60	4.54	272.4	4.54
Physician visits				
General Practitioner				
- Private	360	8.06	2903.23	48.38
- Public				
- Insured	360	1.11	402.82	6.71
- Uninsured	360	3.72	1342.74	22.37
Internist				
- Private	360	13.10	4717.74	78.62
- Public				
- Insured	360	1.34	484.48	8.07
- Uninsured	360	4.48	1614.92	26.91
Endocrinologist				
- Private	360	16.12	5806.45	96.77
- Public				
- Insured	360	1.64	593.35	9.88
- Uninsured	360	5.49	1977.82	32.96

USD; USA dollars.

**Table 5:** Total costs of intervention per patient in USD considering public and private sectors

	**Private Sector**	**Public Sector without insurance coverage**	**Public Sector with insurance coverage**
General Practitioner	217.37	174.84	149.15
Internist	247.61	179.38	150.51
Endocrinologist	265.76	185.43	152.32

USD; USA dollars.

#### Average cost effectiveness analysis

ACER was also calculated for 1.7% improvement in HbA1c as the main outcome per patients with different type visits and lab test tariffs (Table 6).

**Table 6:** ACER per 1% HbA1c reduction per patient considering public and private sectors

	**Private Sector**	**Public Sector without insurance coverage**	**Public Sector with insurance coverage**
General practitioner	127.86	102.85	87.74
Internist	145.65	105.52	88.54
Endocrinologist	156.33	109.08	89.60
Mean	143.28	105.82	88.63

ACER; Average Cost Effectiveness Ratio.

**Figure 1:**
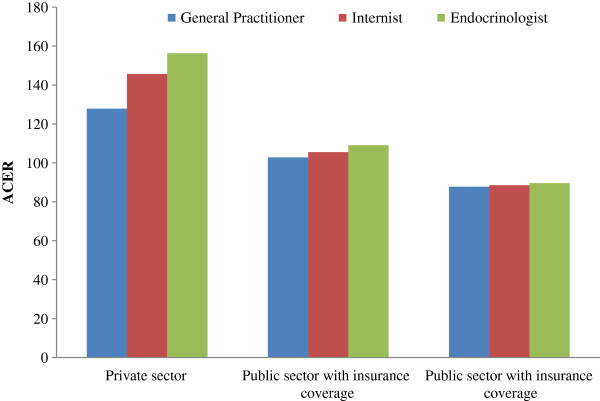
**Sensitivity analysis of effects of costs considering the level of health care professionals in public and private sectors.** ACER; Average Cost Effectiveness Ratio.

As demonstrated in Table 6, the most cost effective alternative is intervention with general practitioner in public sector with insurance coverage. In addition, the sensitivity analysis describes the effects of costs in different types of visit and lab test tariffs on the main results (Figure 1). Results indicate the mean ACER in private sector, public sector without insurance and with insurance coverage are 143.28, 105.82 and 88.63 USD per 1% reduction in HbA1c, respectively. Moreover, mean ACER results in different levels of healthcare professionals are demonstrated in Table 6.

#### Cost saving

In Javanbakht et al. study [23], total annual cost of diabetes related complications and mortality reported to be 879.8 USD (440 USD for 6 month). As shown in Table 7, total complications and mortality cost saving was 154.8 USD. Hence, according to our calculation the public sector with insurance coverage could be chosen as the highest cost saving alternative. 

**Table 7:** Average cost saving for 1.7% HbA1c reduction

**Complications**	**Average cost per patient (USD)**	**Average reduction for 1.7% HbA1c improvement (%)**	**Average Cost saving (USD)**
Microvascular	61.98	62.9	38.98
Cardiovascular disease	106.20	23.8	25.28
Peripheral vascular disease	20.53	73.1	15.00
Ophthalmic	35.00	32.3	11.31
Disability and mortality	216.20	29.75	64.32
Total	439.9		154.89

USD; USA Dollars.

## Discussion

Poor glycemic control results in unfavorable clinical outcomes for people with diabetes. Subsequently, medical costs related to the treatment of diabetic complications will increase. Furthermore, the greatest numbers of people with diabetes are among economically productive age group [2,3]; therefore, it is important to manage the disease in order to ultimately prevent the complications. Considering the findings of the study conducted by Javanbakht et al. [23], T2DM and its complications impose a large economic burden on the individual and health care system in Iran which can mostly be prevented through improved lifestyle and prevention programs. Improved understanding of the economic burden of diabetes also helps health care policy makers for future planning in order to reduce the national burden of diabetes. Thus, in order to manage diabetes and to reduce its associated complications and medical costs, accurate blood glucose measurements are essential [24]. SMBG is widely considered as a key component in management of people with diabetes [25]; thus, we were to investigate the effect of comprehensive patient management using structured SMBG on metabolic control as well as its cost consequence analysis in people with type 2 diabetes in Iran.

We found that structured SMBG contributes significantly in improvement of metabolic outcomes in people with type 2 diabetes. Furthermore, the ACER showed subtractive manner per 1% reduction of HbA1c per patient, from private to public sector considering the state of insurance coverage. In addition, considering the level of health care professionals, a slight reduction in ACER was observed from subspecialty level to the general practitioner level. According to Table 6, the most cost effective hypothesis is the visit by the general practitioners in people with insurance coverage in the public sector and the lowest cost effective hypothesis is attributed to subspecialty visits in the private sector. Many studies have discussed the role of the general practitioners as the “gatekeeper” and vital elements of health care services since restrict direct access to specialists [26]. Kerr et al. reported that only six percent of patients are allowed to self-refer to subspecialists in USA and the “gatekeeper” is required in sixty percent of cases to acquire prescribed pre-authorization [27]. A result of this plan is that patients are being managed by generalists prior to be managed by internists or endocrinologists. So, it would be more effective to implement this result in global health systems. One of the negative effects of imbalanced supply and demand for physician visits is “supply surplus” for general practitioner visits and also “demand surplus” for specialist visits. In diabetes scenario, according to disease complexity, the growing social and economic burden of disease as well as accessibility and affordability of the related health care services are vital. We show that the same metabolic outcome can be achieved regardless of the level of health care professionals.

There are some evidences which are in favor of more effective health seeking behavior and also better use of existing health resources with affordable prices in the public sector [28]. On the other hand, there are some barriers, namely economic issues which put some limitations on the process of patient referral [29]. This study directs cost-effectiveness of SMBG with public sector rather than private sector.

Our study illustrated all SMBG with public sector with insurance coverage are cost beneficial. Differences in ratios are negligible and indicate the importance of insurance to reach equity in health care services. We demonstrated general practitioner visits with insurance coverage as the most affordable option for people with type 2 diabetes.

## Conclusion

The quality of health care provided in public sectors and the insurance coverage are key factors to make health services accessible and also affordable. One of the main limitations in applying SMBG is some uninsured costs of care (for example costs of glucometer, strips and even education). If health care services are to be moved from internists to generalists, additional consideration should be given to have adequate knowledge in generalists and also efficient system resources to supply satisfactory quality of care for people with diabetes. All in all, the 6-month implementation of the comprehensive approach for diabetes management using structured self-monitoring of blood glucose and educational sessions resulted in significant improvement of glycemic status in all subjects in the current study.

## Competing interests

This project was supported by DarmanYab Darou Co., a representative of Roche Diagnostics GmbH (Mannheim, Germany) in Iran.

## Authors’ contributions

This study was conceived by Mohammad E. Khamseh and Mojtaba Malek and its methods developed by all authors. Data were collected by Rokhsareh Aghili and Shahin Yarahmadi analysed by Rokhsareh Aghili and Amir Farshchi. The manuscript was also drafted by Amir Farshchi and Rokhsareh Aghili, critically revised by Mohammad E. Khamseh. All authors read and approved the final manuscript.
